# Annotations

**Published:** 1893-03-25

**Authors:** 


### Annotations.

"Patients Conscientiously Treated."

Delivered by hand, with an embossed crest and motto and the address in bold, red, raised, die-stamped letters, a circular was brought to the door of a friend of the writer's the other day, in the very heart of wealthy London, and the heading of this annotation constituted one of its separate lines and paragraphs. "Patients conscientiously treated." Exactly! Is the author of the circular enough of a literary man to be aware of the significance of his own words? Does he not know that when he thinks it desirable to speak in this way he implies that it is not usual for patients to be conscientiously treated? "Year by year," continues the precious document, "the medical faculty attach more importance to the cure of disease by natural consequences" (*sic*). The gentleman goes on to inform his readers that he "has acquired the knowledge and skill which can only result from long and extensive practice"; the words "long and extensive practice" being swelled out to their utmost serviceable dimensions by the bold use of capitals. Now here is professional advertising in its most dastardly form. In order to increase his own business, this "professional" gentleman basely insinuates that conscientiousness is a quality which is somewhat uncommon in his profession, and that the public had better, therefore, avoid his brethren and trust in him. This is a sample of his "conscientiousness," and if the stock be like the sample, anybody whose wit is more than that of a schoolgirl will take care to avoid it. For our part we have thought it desirable to hand over the circular to the heads of the Medical Protection Society. Perhaps this "conscientious" gentleman may have an opportunity of proving his conscientiousness in another place.

The "Seamy Side" of "Home Sweet Home."

There is something in a title; and under this very modern and somewhat cynical title a leaflet is published by those sanitary reformers who take "plumbing" for their department of practical sanitary activity. Plumbing is to sanitary science what drug dispensing and sick nursing are to the science and art of medicine. The physician may be the most skilful diagnostician in the world, and the most judicious prescriber; but his work will be as futile as a passing breath of wind if the drugs he orders be not of the best and prepared in the best way, or if the nursing be no real nursing at all. Similarly, it is not the scientific sanitarian upon whom in the long run we are obliged to rely, but the plumber who actually puts in our drains and pipes and sanitary contrivances, and who, after putting them in, buries them deep under-ground out of our sight. He is the real person in whom, perforce, our trust is to be placed. Let him but be a man of character and capacity, and we shall be absolutely "alright." Let him be a fool or a knave, and he may easily be either the one or the other, without being extraordinarily singular, and then we may be so "wrong" that nothing but illness or a death or two can in future make us "right." Must we not then "beware of the plumber?" In this connection Mr. Kaye Parry, whose sanitary work we have noticed before in The Hospital, delivered a most interesting and valuable lecture at Cork a few days ago under the presidency of the Deputy Mayor, Mr. Alderman Sheehan. Mr. Parry said we were sometimes told that "the present sanitary craze was a humbug invented by persons who desired to make money out of the fears of the nervous and the ignorant. But, such criticism notwithstanding, it could be clearly shown that there was a real connection between the state of our houses and the state of our health. In every town where a proper system of drainage and water supply had been carried out the death-rate had been substantially reduced thereby." During the past few years Mr. Kaye Parry has himself inspected more than 1,500 buildings, and in hundreds of cases he has been able to trace sickness and death to defects in plumbing and drainage, or to water pollution. These are hard facts; and the facts of sanitary science are just as definite and real as the calculations of arithmetic. The "answer" of a sanitary problem "comes out" as infallibly as the "answer" of a sum in "practice" or "rule of three." When we are all really educated we shall know these things, and the knowledge will make us and our children a great deal healthier and a great deal happier.

The Children's Teeth.

For real health and comfort it is as necessary to have sound teeth as to have sound lungs or a sound brain. Mr. Denison Pedley, F.R.C.S., dental surgeon to the Evelina Hospital for Sick Children, has recently undertaken a dental examination of the children in three of the principal metropolitan Poor Law schools. The number of children examined amounted to 3,145, and the number of separate teeth to 70,000. The statistics therefore cover a very considerable area of observation. A tabular statement published by Mr. Pedley reveals a great many curious and interesting facts. For instance, it was found that, of the whole 3,145 children examined, only 707 had quite sound teeth. That is to say, rather less than one in every five of that particular class of children were blessed with teeth that were healthy. The table of statistics may be divided into "toothache periods." The period of "maximum toothache," as we might perhaps have expected, is from the age of seven to the age of twelve; it begins with the second dentition. The period of "minimum toothache" is from the age of twelve to the age of fifteen; that is, after the second dentition is well established. The point of greatest practical importance is that a great many teeth go wrong and inflict permanent injury as well as disfigurement upon children, which might easily be prevented by frequent inspections of the mouth. For example, in 110 children examined at the age of four, there were found no fewer than 290 teeth which required filling or extracting. But many people imagine that the teeth of children so young, being their first teeth, require no attention of any kind. The mistake is most serious, and may lead to life-long injury. In 340 children, examined at the age of nine, there were found the extraordinary number of 1,143 unsound teeth—more than three in the mouth of every child examined. As many as 833 of these were found capable of cure by filling, whilst no more than 310 required extraction, of which eight only were permanent teeth. These are but a sample of Mr. Denison Pedley's facts. But they are most instructive. There are reasons for believing that the teeth of the class of children examined are rather above than below the average for all classes. It is said, indeed, that as we ourselves ascend in the social scale our teeth descend in quality. The obvious conclusion is that our children's mouths ought to be periodically inspected by competent and honest dentists. We ought no more to expose children to risks of dyspepsia, disfigurement, and other injurious consequences of bad teeth than to bad drainage, or to a climate of consumption.

